# Profil épidémiologique et clinique des tentatives de suicide chez l'enfant et l'adolescent en Tunisie en post révolution

**DOI:** 10.11604/pamj.2019.32.204.15477

**Published:** 2019-04-26

**Authors:** Fatma Charfi, Azza Harbaoui, Afef Skhiri, Zeineb Abbès, Ahlem Belhadj, Soumaya Halayem, Asma Bouden

**Affiliations:** 1 Service de Pédopsychiatrie, Hôpital Mongi Slim de La Marsa, Sidi Daoud 2046, Tunis, Tunisie; 2 Faculté de Médecine de Tunis, Université Tunis El Manar, Tunis, Tunisie; 3 Service de Pédopsychiatrie, Hôpital Razi, rue des Orangers, 2010 La Manouba, Tunisie; 4 Institut National de la Santé, 5-7 rue Khartoum, 1002 Tunis, Tunisie

**Keywords:** Tentative de suicide, enfant, adolescent, troubles mentaux, Suicide attempt, child, adolescent, mental disorders

## Abstract

**Introduction:**

Les conduites suicidaires sont en augmentation en Tunisie et touchent une population de plus en plus jeune. Le but de notre étude était de décrire le profil sociodémographique et clinique des tentatives de suicide chez l'enfant et l'adolescent.

**Méthodes:**

Cette étude était transversale et descriptive, incluant 50 suicidants, recrutés au service de pédopsychiatrie de l'Hôpital Razi de la Manouba et dans deux services de réanimation et de pédiatrie de Tunis, entre juillet 2012 et juin 2013. Ont été relevés les facteurs sociodémographiques et cliniques, les antécédents de maltraitance, la scolarité, les caractéristiques de la TS, l'intentionnalité suicidaire évaluée par le Suicide Intent Scale, et les troubles psychopathologiques à l'aide du Mini-International Neuropsychiatric Interview.

**Résultats:**

Le sex-ratio était de 0,56, la moyenne d'âge était de 12,4 ans avec des extrêmes de 7 à 16 ans. Un échec ou un fléchissement scolaire a concerné 86% des suicidants. Dans 38% des cas il s'agissait de récidive; des antécédents d'automutilations ont été retrouvés dans les mêmes proportions. Un contexte de maltraitance a été signalé dans 46% des cas. L'ingestion médicamenteuse était le moyen le plus fréquent, les psychotropes étant les plus représentés. Une différence significative entre le genre a été retrouvée dans le recours aux moyens suicidaires, ainsi les garçons ont eu davantage recours aux moyens physiques (p=0,04) et les filles aux intoxications (p=0,001). L'intentionnalité suicidaire était élevée dans 44%. Un épisode dépressif majeur et le trouble de l'adaptation étaient les troubles les plus fréquemment retrouvés dans respectivement 58% et 24% des cas.

**Conclusion:**

Les troubles dépressifs et la maltraitance se dégagent comme des facteurs de risque des TS chez les enfants et les adolescents, ces facteurs doivent être pris en considération dans les stratégies de prévention du suicide dans cette population.

## Abstract (en)

**Background:**

Suicidal behaviors are increasing in Tunisia at younger and younger ages. This study aims to describe the sociodemographic and clinical profile of suicide attempts in children and adolescents.

**Methods:**

We conducted a cross-sectional and descriptive study, including 50 suicidal individuals, recruited in the Child Psychiatry Department at the Razi Hospital in Manouba and in two Departments in Tunisia (Intensive Care and Reanimation Department and Department of Pediatrics) between July 2012 and June 2013. We identified the socio-demographic and clinical factors, the histories of abuse, subjects' education, the characteristics of the attempted suicides (TS), suicidal ideation assessed by the Suicidal Intent Scale and psychopathological disorders diagnosed using the Mini-International Neuropsychiatric Interview.

**Results:**

The sex-ratio was 0.56, the average age was 12.4 years ranging from 7 to 16 years. School failure or dropout were found in 86% of suicidal individuals. In 38% of cases suicidal behavior was recurrent; the subjects had a history of self-mutilation to the same extent. A context of abuse was reported in 46% of cases. Drug ingestion was the most common means of suicide, psychotropic drugs were the most common substances used. There was a significant gender difference in the use of the means of suicide: the boys mainly used physical means (P=0.04) while the girls poisoning (P=0.001). Suicidal intent was high in 44% of cases. A major depressive episode and adjustment disorder were the most common disorders found (58% and 24% of cases respectively)

**Conclusion:**

Depressive disorders and abuse are risk factors of TS in children and adolescents, these factors should be considered when implementing suicide prevention strategies for this population.

## Introduction

Le suicide représente la seconde cause de mortalité chez les 15-29 ans dans le monde d'après l'OMS [1]. En Tunisie, les données récentes ont montré une augmentation de l'incidence du suicide après la révolution de 2011, avec un profil différent en comparaison avec la période antérieure à 2011 [2]. La proportion des jeunes de moins de 19 ans décédés par suicide en 2015 représentaient 13,4% des cas selon les statistiques du Ministère de la Santé; et dans une étude menée dans une région du centre de la Tunisie, une augmentation de la mortalité par suicide chez les mineurs a été objectivée entre 2009 et 2015 [3, 4]. Quant aux tentatives de suicide (TS), ce sont des comportements préoccupants par le risque de morbidité et le risque de récidive qu'elles représentent, un tiers des jeunes décédées par suicide avaient des antécédents de TS et de ce fait elles constituent un facteur de risque majeur d'évolution fatale [5]. Ces conduites suicidaires constituent une préoccupation majeure de santé publique et interrogent les cliniciens devant leur complexité et la diversité des facteurs de risque. Parmi ces facteurs, on retrouve les troubles mentaux, surtout les troubles de l'humeur, et les facteurs d'adversité psycho-sociaux tel que la maltraitance, un environnement conflictuel et les évènements de vie négatifs [6]. Ainsi, la compréhension de ces facteurs permet de mieux cibler les actions de prévention. Le processus suicidaire est de déroulement rapide chez l'adolescent avec un caractère souvent impulsif. Pour ce qui est des conduites suicidaires chez l'enfant de moins de 13 ans, Mishara souligne que même si la compréhension et la représentation de la mort n'est pas encore achevée, ceci n'empêche pas la survenue chez l'enfant d'un acte suicidaire avec l'intention de se donner la mort [7]. Les données de la littérature sur les TS de l'enfant sont beaucoup plus rares et ont mis en exergue le possible recours aux moyens violents, l'importance des facteurs socio-environnementaux, et le risque de récidive ultérieur notamment à l'âge adulte [6, 8, 9]. Dans le contexte tunisien, très peu d'études ont concerné les comportements suicidaires chez l'enfant et l'adolescent, ce sont essentiellement des études rétrospectives [10-12]. Notre étude s'inscrit dans les suites de ces travaux et avait pour objectif d'étudier le profil sociodémographique et clinique d'une population d'enfants et d'adolescents suicidants. Ainsi, nous nous sommes intéressés à évaluer les troubles psychopathologiques et les facteurs d'environnement ainsi que l'intentionnalité suicidaire dans cette population.

## Méthodes

Il s'agit d'une étude descriptive et transversale menée auprès d'une population clinique d'enfants et d'adolescents suicidants durant la période du 1^er^ juillet 2012 au 30 juin 2013. Une autorisation du comité d'éthique local a été obtenue.

**Population de l'étude:** Ont été inclus dans notre étude les enfants et les adolescents qui ont consulté suite à une TS dans les trois structures de soins suivantes: 1) Tous les suicidants qui ont consulté le service de pédopsychiatrie de l'hôpital Razi à La Manouba durant la période de l'étude, seul service de psychiatrie de l'enfant et de l'adolescent de la capitale et du nord de la Tunisie jusqu'à fin 2013. 2) Les enfants hospitalisés aux services de pédiatrie de l'Hôpital d'enfants de Tunis, et les adolescents âgés de moins de 17 ans hospitalisés au Centre d'Assistance Médicale Urgente de Tunis, pour lesquels un avis pédopsychiatrique a été demandé lors de la période de l'étude. Ont été exclus de l'étude les enfants ayant un retard mental. De même que les suicidants dont la TS remonte à plus de 48 heures: sauf les cas présentant une altération de l'état neurologique, ces sujets ont été examinés après reprise d'état de conscience. L'évaluation des suicidants a été faite après information des parents du but de l'étude et l'obtention de leur consentement, par un résident en pédopsychiatrie affecté au service de pédopsychiatrie de l'Hôpital Razi durant la période de l'étude. Ces entretiens étaient réalisés dans les suites de l'entretien psychiatrique initial, réalisé dans un contexte d'urgence et dont le but était de décider de la prise en charge et d'évaluer le degré d'urgence et le potentiel de récidive.

**Instruments de mesure et d'évaluation:** Nous avons utilisé une fiche préétablie conçue pour l'étude pour les caractéristiques sociodémographiques, l'évaluation de l'environnement, la scolarité et les caractéristiques de la TS. Elle a comporté des items concernant le statut des parents, les relations avec les pairs, la présence actuelle et les antécédents de maltraitance, les antécédents médicaux et psychiatriques personnels et familiaux du patient ainsi que la symptomatologie psychiatrique précédant le passage à l'acte. Concernant le niveau socioéconomique, ce dernier a été apprécié par le clinicien en tenant compte de la profession des parents, de la taille de la famille et des conditions de vie. Ont été également précisées les caractéristiques de la TS, les circonstances de son déroulement, le cadre temporo-spatial et le moyen utilisé. Deux questions ont été enfin posées sur l'accès à l'information concernant le suicide et les sources de celles-ci. L'évaluation des troubles psychopathologiques a été réalisée par la version courte et traduite en arabe du *Mini International Neuropsychiatric Interview for Children and Adolescents* (MINI-KID) [13]. Le MINI-KID est un entretien diagnostique semi-structuré destiné à évaluer les épisodes actuels et passés psychopathologiques chez l'enfant et l'adolescent de l'axe I du DSM-IV (la 4^ème^ version du manuel diagnostic et statistique des troubles mentaux) [14]. C'est un hétéro questionnaire, qui se divise en 16 modules qui explorent les troubles de l'humeur, les troubles anxieux, l'état de stress post traumatique, l'abus et la dépendance aux substances, les troubles du comportement alimentaires, l'hyperactivité déficit de l'attention, le trouble oppositionnel, les troubles des conduites et les psychoses. Il n'explore pas les troubles spécifiques du développement ni les troubles de l'attachement. L'évaluation de l'intentionnalité suicidaire a été réalisée à l'aide du questionnaire *Suicide Intent Scale (SIS)*, traduit en arabe pour cette présente étude, mais non validé. Il s'agit d'un hétéro-questionnaire, mis au point par Beck en 1974, revu par Pierce en 1977 qui est destiné à évaluer l'intensité du désir de mort du patient au moment de sa tentative de suicide et prédit le risque suicidaire [15, 16]. La version utilisée est divisée en trois sections: les circonstances de la tentative de suicide (six questions), les propos rapportés par le patient (*self report*) (quatre questions) et deux questions sur la létalité évaluée par le médecin. Le score total d'intentionnalité suicidaire varie de 0 à 25: intentionnalité faible: 0-3; intentionnalité moyenne: 4-10; intentionnalité élevée: 11-25.

**Analyse statistique:** La saisie des données ainsi que l'analyse statistique ont été effectuées par le logiciel SPSS version 16.0. Nous avons comparés deux sous-groupes, les enfants de moins de 13 ans, et ceux ayant 13 ans et plus. Le test Khi 2 a été utilisé pour la comparaison des pourcentages des variables qualitatives dichotomiques et catégorielles et le seuil de signification a été fixé à 5%.

## Résultats

**Caractéristiques sociodémographiques et familiales:** Les résultats sont présentés au Tableau 1. Notre population était constituée de 50 suicidants. Soixante-dix-huit pourcent des patients étaient recrutés au service de Pédopsychiatrie de l'Hôpital Razi et 22% des cas ont été initialement examinés dans les autres structures de soins où ils étaient admis (Hôpital d'enfants de Tunis et Centre d'Assistance Médicale Urgente de Tunis). Le sexe ratio (G/F) était de 0,56. La moyenne d'âge de notre population était de 12,4 ans avec des extrêmes allant de 7 à 16 ans, 42% des cas étaient âgés de moins de 13 ans et 58% de 13 à 16 ans. Dans notre série, 38% étaient les benjamins de leur fratrie, 28% étaient les cadets, 24% l'aîné et 10% étaient l'enfant unique du couple parental. Près de la moitié des cas (48%) vivaient dans un contexte de séparation ou de divorce des parents, et dans 12% des cas, un climat de violence conjugale était retrouvé. La relation avec les pairs était jugée comme satisfaisante dans 68% des cas et conflictuelle dans 32% des cas. Concernant le niveau socioéconomique, il était jugé comme élevé dans 24% des cas, moyen dans 54% des cas et bas dans 22% des cas. Sur le plan scolaire, on note que la majorité des sujets étaient scolarisés (98%). Dans 86% des cas, le rendement scolaire était perturbé durant l'année précédant la consultation avec la présence d'un fléchissement ou d'un échec scolaire.

**Tableau 1:** Caractéristiques sociodémographiques et cliniques des suicidants, population totale et comparaison en fonction de l’âge (<13 ans et ≥13 ans)

		Population des suicidants N=50		Age <13 ans N=21		Age ≥13 ans N=29		*p*
		%	N	%	N	%	N	
Genre	Fille	64,0	32	61,9	13	65,5	19	0,79
	Garçon	36,0	18	38,1	8	34,5	10	
Niveau socioéconomique	Bas	22,0	11	4,8	1	34,5	10	0,03
	Moyen	54,0	27	71,4	15	41,4	12	
	Elevé	24,0	12	23,8	5	24,1	7	
Situation familiale	Séparation, divorce des parents	48,0	24	52,38	11	44,83	13	0,59
	Famille avec deux parents présents	52,0	26	47,62	10	55,17	16	
	Autre situation	0	0	0	0	0	0	
Violence familiale	Oui	12,0	6	4,76	1	17,24	5	0,18
	Non	88,0	44	95,24	20	82,7	24	
Relation avec les pairs	Bonne	68,0	34	81,0	17	58,6	17	0,1
	perturbée	32,0	16	19,0	4	41,4	12	
Antécédents familiaux de troubles psychiatriques	Oui	66,0	33	57,1	12	72,4	21	0,28
	Non	34,0	17	42,9	9	27,6	8	
Antécédents familiaux de tentative de suicide	Oui	22,0	11	9,5	2	31,0	9	0,07
	Non	78,0	39	90,5	19	69,0	20	
Antécédents personnels somatiques	Oui	22,0	11	23,8	5	20,7	6	0,79
	Non	78,0	39	76,2	16	79,3	23	
Antécédents personnels psychiatriques	Oui	24,0	12	19,0	4	27,6	8	0,51
	Non	76,0	38	81,0	17	72,4	21	
Antécédents personnels de tentative de suicide	Oui	38,0	18	49 ,6	10	20,7	9	0,79
	Non	62,0	31	52,4	11	69,0	20	
Antécédents personnels d’auto-mutilation	Oui	36,0	18	38,1	8	34,5	10	0,79
	Non	64,0	32	61,9	13	65,5	19	
Maltraitance	Oui	46,0	23	23,8	5	62,07	18	0,007
	Non	54,0	27	76,19	16	37,9	11	
Type de maltraitance	Physique	69 ,5	16	40,0	2	77,78	14	0,14
	Sexuelle ou psychologique	30,4	7	60,0	3	22,2	4	
Milieu de maltraitance	Familial	78,2	18	19,0	4	48,3	14	0,7
	Scolaire	21,7	5	4,8	1	13,8	4	
Moyens utilisés pour la TS	Médicaments et toxique	80,0	40	85,7	18	75,9	22	0,11
	Strangulation ou pendaison	10,0	5	0	0	17,2	5	
	Prise de risque	10,0	5	14,3	3	6,9	2	
Moyen suicidaire accessible	Oui	72,0	36	71,4	15	72,4	21	0,94
	Non	28,0	14	28,6	6	27,6	8	
Existence d'une symptomatologie psychiatrique précédant la TS	Oui	80,0	40	71,4	15	86,2	25	0,22
	Non	20,0	10	28,6	6	13,8	4	
Répercussions organiques de la TS	Oui	66,0	33	57,1	12	72,4	21	0,28
	Non	34,0	17	42,9	9	27,6	8	
A entendu parler de suicide auparavant	Oui	76,0	38	85 ,7	18	69,0	20	0,17
	Non	24,0	12	14,3	3	31,0	9	
Trouble psychopathologique (MINI Kid)	Trouble de l'adaptation avec anxiété et dépression	24,0	12	42,9	9	10,3	3	0,007
	Autre ou pas de trouble caractérisé	76,0	38	57,1	12	88,7	26	
Score SIS	Risque faible ou moyen	56,0	28	71,4	15	44,8	13	0,06
	Risque élevé	44,0	22	28,6	6	55,2	16	

**Antécédents personnels et familiaux:** Les antécédents familiaux psychiatriques ont été retrouvés dans les 2/3 des cas, avec majoritairement des troubles de l'humeur dans 52% des cas, et des antécédents familiaux de TS ont été relevés dans 22%. Des antécédents personnels de pathologies organiques chroniques ont été notés dans 22% des cas. Il s'agissait essentiellement d'épilepsie (8%), d'asthme (4%) et de diabète juvénile (6%). Le quart des patients avaient des antécédents psychiatriques connus: troubles de l'humeur (8%), trouble déficit de l'attention avec hyperactivité (8%), trouble des conduites (6%) et troubles anxieux (2%). Pour ce qui est des antécédents de comportements suicidaires, 38% avaient déjà fait au moins une TS (avec 8% des cas plus d'une TS), et des antécédents d'automutilation ont été retrouvés dans 36% des cas.

**L'exposition à la maltraitance:** Parmi nos patients, 46% ont déclaré avoir subi des maltraitances, il s'agissait le plus souvent de maltraitance physique (32%). Deux patients ont subi des violences sexuelles (4%) et 10% ont été victimes de maltraitance « psychologique » répétée à type de: rejet, humiliation ou intimidation (y compris en milieu scolaire). Le milieu familial a été incriminé dans 36% des cas et le milieu scolaire dans 10% des cas.

**Caractéristiques cliniques de la TS et le contexte de sa survenue:** Le moyen suicidaire le plus fréquent était l'ingestion médicamenteuse qui a concerné 78% des cas (n=39), l'intoxication était poly médicamenteuse dans 26% des cas (n=13), les molécules psychotropes étaient les médicaments les plus utilisés, dans 42% (n=21). Les suicidants ont eu recourt à la tentative de pendaison ou la strangulation dans 10% des cas (n=5), les autres moyens physiques dans 10% des cas (défénestration et tentative de précipitation devant un véhicule) et un cas d'ingestion de raticide; avec une différence significative selon le genre. Les garçons ont eu plus fréquemment recourt aux moyens physiques (p=0,04) et les filles aux moyens médicamenteux (p=0,001). Concernant la répartition des TS selon le mois de survenue et selon l'heure de survenue, la fréquence des tentatives de suicide était plus élevée au cours des mois de mars (9 cas, 18%) et d'avril (8 cas, 16%) ; et survenait davantage en fin de journée: 16% à 19h, 20% à 20h. Un évènement précédent la survenue de la TS a été retrouvé dans 62% des cas, il s'agissait: de conflit familial (26%), de conflit avec les pairs et/ou conflit en milieu scolaire (12%) ou d'une rupture sentimentale (24%). L'évaluation de l'intentionnalité suicidaire par la SIS a retrouvé un niveau d'intentionnalité faible dans 14%, moyen dans 42% et élevé dans 44% des suicidants. Des symptômes précédents le geste suicidaire ont été retrouvés dans 80%, il s'agissait de symptômes à type d'irritabilité, de retrait, d'anxiété et troubles du comportement. Ces symptômes étaient présents depuis plus de trois mois avant la TS dans 72% des cas. Soixante-six pour cent des suicidants avaient des répercussions somatiques dans les suites de leur TS, ayant nécessité une prise en charge médicale, le tiers d'entre eux (22%) a nécessité une hospitalisation en pédiatrie ou en réanimation (cf inclusion des patients).

**Les diagnostics psychopathologiques:** Les troubles psychopathologiques évalués par le MINI-KID dans notre population sont représentés dans le Tableau 2. Une corrélation significative entre l'existence d'un trouble psychiatrique et une intentionnalité suicidaire moyenne à élever au SIS a été retrouvée (p=0,002). Au terme de l'entretien psychiatrique et de l'évaluation clinique, la prise en charge était d'emblée ambulatoire dans 58% des cas et 42% étaient hospitalisés en pédopsychiatrie.

**Tableau 2:** Les diagnostics psychopathologiques établis par MINI-Kid

Diagnostic	N=50	%
Episode dépressif majeur	29	58,0
Trouble de l’adaptation avec à la fois anxiété et humeur dépressive	12	24,0
Trouble des conduites	3	6,0
Abus de substance	3	6,0
Aucun trouble psychiatrique	3	6,0

**L'accès à l'information concernant le suicide:** Dans notre population, 76% des cas ont déclaré avoir déjà entendu parler de suicide et avaient une connaissance sur le sujet. Les sources de ces informations ont été essentiellement la famille dans 32% des cas, les médias et les réseaux sociaux dans 22% et le milieu extrafamilial dans 16% des cas.

**La comparaison des sous-groupes en fonction de l'âge:** La comparaison des deux groupes en fonction de l'âge n'a pas montré de différences significatives sur plan des caractéristiques sociodémographiques, les antécédents médico-psychiatriques, les moyens suicidaires, l'accès à l'information sur le suicide et les scores de la SIS (Tableau 1). En revanche, sur le plan clinique, le trouble de l'adaptation avec à la fois anxiété et humeur dépressive était significativement plus fréquent chez les enfants âgés de moins de 13 ans comparés aux plus âgés (p=0,007) (Tableau 1). De plus les suicidants de plus 13 ans étaient davantage exposés à la maltraitance que ceux de moins de 13 ans (p=0,007).

## Discussion

**Les caractéristiques sociodémographiques et familiales:** Une nette prédominance féminine retrouvée dans notre série rejoint les données de la littérature [10, 12, 17-20]. En effet, les filles dès la pré adolescence ont plus d'idées suicidaires et font plus de TS que les garçons. La moyenne d'âge de notre population était de 12,4 ans, elle a été en partie déterminée par la limite d'âge de la structure de soin où s'est déroulée l'étude qui est de 16 ans. Une étude réalisée dans la même structure sur l'évolution des TS entre 2005 et 2015 a montré un rajeunissement des TS à partir de 2013 ce qui plaide en faveur d'une augmentation des TS chez l'enfant dans le contexte tunisien après 2011 [12]. Des études réalisées en population jeune, trouvent une moyenne d'âge comparable à celle de notre échantillon, ainsi dans une étude Marocaine, l'âge moyen était de 12,5 ans [18]. Dans une étude française menée sur 517 suicidants âgés de moins de 15 ans admis aux services des urgences pédiatriques l'âge moyen était de 14 ans et celui du plus jeune suicidant était de 7 ans [19]. Le rajeunissement de la population des suicidants peut être lié à l'exposition de plus en plus fréquente des enfants aux facteurs d'adversité psycho-sociaux et aux adversités vécues par le couple parental et la famille.

Un contexte de divorce ou de séparation parentale a été observé dans près de un cas sur deux, ce qui est largement supérieur aux données de la population générale tunisienne de la même année de la réalisation de la présente étude [21]. Une étude marocaine a retrouvé des résultats similaires [18]. De façon générale, c'est le manque de cohésion familial qui a été suggéré comme ayant un rôle dans l'émergence des conduites suicidaires [22]. Le profil socioéconomique des familles des suicidants dans notre série, était considéré comme moyen dans la moitié des cas et faible dans 22%, est similaire à celui de la population qui consulte habituellement ce service [23]. Même si le lien entre faible niveau socioéconomique et TS a été suggéré en population adolescente avec des méthodologies robustes, ce facteur a suscité des controverses [6, 24]. Les difficultés et l'échec scolaire retrouvés dans une proportion élevée (86% des suicidants) plaident en faveur de l'importance de ce facteur de risque, et de fait les enfants en échec scolaire sont moins résilients face à l'adversité et se projettent moins dans l'avenir que leurs pairs qui réussissent dans leur scolarité. Une association entre les difficultés scolaires et les conduites suicidaires a été rapportée par certaines études [10, 25]. Ils constituent ainsi un des facteurs à prendre en considération pour la prévention du risque suicidaire.

**La maltraitance:** Un contexte de maltraitance a été signalé dans près de la moitié des cas émanant le plus souvent du milieu familial, et retrouvé en majorité chez les sujets âgés de 13 ans et plus. L'exposition à la maltraitance est un facteur de risque de suicide bien documenté dans la littérature, tant pour les TS que pour le suicide accompli [26, 27]. Dans une étude menée par Séguin *et al*, sur les trajectoires de vie de jeunes suicidés, l'exposition à la violence sexuelle ou physique a été retrouvée de manière significative dès la petite enfance en comparaison aux sujets témoins [5]. Plusieurs auteurs s'accordent sur le fait que l'abus sexuel est un facteur fragilisant pour la santé mentale du jeune en général et un facteur de risque spécifique pour les comportements suicidaires [27]. Une méta-analyse qui a inclus des études longitudinales et un total de 8733 participants, a montré que les antécédents d'abus sexuels sont associés à la survenue de tentatives de suicide, et ce après avoir contrôlé les autres facteurs de risques génétiques et environnementaux [28]. Dans notre série, ce facteur a été rarement retrouvé (seulement 4% des suicidants ont été victimes d'abus sexuel), ce faible taux peut être expliqué par la taille réduite de notre échantillon, mais aussi par la réticence des jeunes à aborder cette question, qui reste un sujet tabou dans le contexte tunisien. De plus, la première évaluation clinique de l'enfant et du jeune au décours de sa TS est un moment de grande détresse psychologique ce qui rend l'abord de la question de la maltraitance, spécifiquement l'abus sexuel, délicat.

**Les antécédents pathologiques personnels et familiaux:** Les antécédents familiaux psychiatriques ont été retrouvés dans les 2/3 des cas, avec essentiellement des troubles de l'humeur, et des antécédents familiaux de TS ont été relevés dans 22% des cas. Ces résultats sont confortés par d'autres études, la pathologie mentale des parents est considérée comme un facteur de risque de suicide chez leurs enfants [25]. De même, les comportements suicidaires dans l'entourage augmentent ce risque. Une pathologie organique chronique a été notée chez près d'un quart de nos patients. Des données de la littérature indiquent que la présence d'une pathologie somatique associée augmentait le risque de suicide chez les patients souffrant d'une comorbidité psychiatrique [29]. Quant aux antécédents de pathologies pédopsychiatriques, ils ont été retrouvés chez le quart de nos patients. Ce résultats est inférieur à ce qui est retrouvé dans d'autres études, Berthod *et al*rapportent un suivi pédopsychiatrique préexistant chez des enfants suicidants dans 69% des cas [8]. Cette différence peut être expliquée par le manque d'offre de soins pédopsychiatriques dans le contexte tunisien, le service de pédopsychiatrie de l'Hôpital Razi était la seule structure pour le nord de la Tunisie avant 2013 [23]. Pour ce qui est des antécédents personnels de TS et des antécédents d'automutilations, ils ont été retrouvés dans plus du tiers de nos patients. Il a été établi par les études longitudinales de suivi après une TS, ainsi que les études d'autopsie psychologique sur les jeunes suicidés, que les TS sont prédictives de passage à l'acte suicidaire ultérieur et ce facteur de risque serait indépendant des autres variables [5, 6, 30]. Quant aux automutilations, certains auteurs les considèrent comme faisant partie des comportements suicidaires, ayant des facteurs de risque communs; pour ces auteurs, automutilations et suicides aboutis seraient les extrêmes d'un même spectre [31].

**Le geste suicidaire:** L'étude du déroulement temporel a montré que le plus grand nombre de TS étaient survenus au cours des mois de mars et avril avec respectivement neuf et huit cas. Une étude menée sur une population de 2393 suicidants tchèques âgés entre 9 à 18 ans a montré que 31,3% des TS se déroulaient au printemps (mars, avril, mai) avec une différence significative par rapport aux autres saisons [20]. Une étude française menée auprès d'enfants suicidants a enregistré un pic au mois de juin, concomitant à la fin de l'année scolaire qui est un moment de bilan [8]. Des hypothèses explicatives ont été avancées notamment les facteurs climatiques et les rythmes scolaires. Dans notre travail, les mois de mars et avril, correspondait en Tunisie à la fin du 2^ème^ trimestre de l'année scolaire et constituait de ce fait une période de stress important. A un niveau circadien, on a observé les pourcentages les plus élevés entre 14 heures et 20 heures, nos résultats rejoignent l'étude tchèque, qui a retrouvé 40,7% des TS survenue entre 18 heures et minuit, 30,1% entre 12 heures et 18 heures ce qui diffère des horaires de survenue en populations adultes majoritairement dans la tranche horaire 22 heure- 7 heure [20]. Soixante-dix-huit pourcent des suicidants dans notre étude ont eu recours à l'intoxication médicamenteuse volontaire, significativement chez les filles. Ceci a été habituellement retrouvé dans les études tunisiennes et dans le monde [9, 10, 18, 19]. Les psychotropes et les médicaments contenant du paracétamol sont les plus fréquemment incriminés [20]. Les moyens violents, quant à eux, ont été plus rares et observés de manière significative chez les garçons, conformément aux données de la littérature, les garçons sont plus à risque de suicide abouti que les filles [5, 8]. Certains auteurs ont souligné que les enfants utilisant des moyens violents étaient plus jeunes que ceux utilisant des moyens non violents [8]. Notons enfin, qu'il a été observé dans le contexte tunisien après la révolution tunisienne de 2011 le recours plus fréquent aux moyens violents chez les jeunes tunisiens [12]. L'intentionnalité suicidaire évaluée par le SIS était moyenne dans 42% et élevée dans 44% des cas, et ce sans différence en fonction de l'âge entre les moins de 13 ans et les 13 ans et plus, ce qui dénote d'un degré de préméditation, de planification de la TS et/ou d'un désir de mort élevé y compris chez l'enfant suicidant. Ceci n'a pas été retrouvé dans une étude réalisée auprès des enfants de moins de 12 ans, où l'intentionnalité suicidaire était moindre, suggérant des différences dans la genèse du passage à l'acte et la représentation de la mort en fonction de l'âge [9]. Cette différence peut être expliquée par le recrutement de nos patients, se faisant essentiellement dans l'institution psychiatrique, et de ce fait pouvant surreprésenter des situations de TS « grave ».

**Les troubles psychopathologiques:** La fréquence importante des pathologies psychiatriques, en particulier la dépression, dans notre série est expliquée par la nature du recrutement des patients où s'est déroulée l'étude, puisque jusqu'à une période récente, l'examen pédopsychiatrique n'était pas demandé de manière systématique par les services de pédiatrie auprès des suicidants, seulement dans les situations jugées comme problématique avec un risque de récidive élevé. De plus, la situation du service de pédopsychiatrie en hôpital psychiatrique, avec la stigmatisation de cet hôpital, implique que les TS « moins grave » faisaient moins l'objet de consultation dans cette institution. Quoiqu'il en soit, les troubles psychiatriques ont été considérés comme un des principaux facteurs de risque proximaux des comportements suicidaires, et la dépression est la pathologie la plus fréquemment incriminée. Dans notre population, le trouble dépressif majeur a été retrouvé dans 58% des cas suivi par le trouble de l'adaptation dans 24%, pour ce dernier, significativement chez les enfants de moins de 13 ans. Dans l'étude de Consoli et al, 64% des adolescents hospitalisés en pédopsychiatrie pour TS avaient un diagnostic d'épisode dépressif majeur, et l'étude de suivi après 6 mois a montré que le trouble dépressif majeur et le désespoir constituaient des facteurs de risque de suicidalité [32]. En effet, la dépression associe désespoir, souffrance psychique insupportable, pessimisme, mauvaise estime de soi et impulsivité, tous ces facteurs augmentent le potentiel suicidaire. Par ailleurs, le trouble de l'adaptation a été majoritairement rapporté dans les études de TS de l'enfant [8], ce qui rejoint nos résultats, la fréquence élevée de ce trouble est le reflet de la fréquence des TS chez les enfants en réaction aux facteurs environnementaux entrainant un débordement des capacités adaptatives face à ces situations. Les TS chez l'enfant pré-pubère ont été d'ailleurs considérées comme une « manifestation d'inadaptation » devant une détresse extrême dans cette tranche d'âge non encore parvenue à tolérer la frustration [8]. Quant au trouble des conduites et l'abus de substance, ils ont été plus rarement retrouvés dans notre population en comparaison aux données de la littérature [6]. Enfin, dans notre série, nous n'avons pas porté de diagnostic de trouble de la personnalité, l'âge limite étant de 16 ans, et l'instrument diagnostic utilisé, le MINI-KID, n'évalue pas cette catégorie diagnostique. Nous n'avons pas non plus retrouvé de cas de schizophrénie ni de trouble bipolaire. Ceci pourrait être expliqué par l'âge jeune des sujets inclus de notre échantillon, ainsi que la faible taille de notre population.

Dans notre travail, une hospitalisation en pédopsychiatrie a concerné 42% des cas. L'hospitalisation était de quelques jours et avait pour but une meilleure évaluation psychosociale et une prise en charge initiale intensive centrée sur la psychothérapie individuelle et l'accompagnement familial. Selon des données françaises, la durée de l'hospitalisation diminue avec l'âge, et elle devrait être systématique après toute TS [8]. Dans le contexte tunisien, l'hospitalisation en pédiatrie est déterminée par les indications somatiques et il existe très peu de services d'hospitalisation en pédopsychiatrie (deux structures à l'échelle nationale), ainsi l'alternative à l'hospitalisation est le suivi en ambulatoire avec des rencontres rapprochées. Par ailleurs, un signalement est souvent réalisé après une TS de l'enfant au délégué de la protection de l'enfance, ce signalement est systématique quand le geste est grave et impose une hospitalisation en réanimation. Il aboutit à des mesures de protection, surtout que la maltraitance et les autres facteurs psychosociaux sont souvent retrouvés. Les services de protection de l'enfance en Tunisie ont enregistré une augmentation importante des signalements de TS et de cas maltraitance ces dernières années [33]. La prise en charge est de ce fait globale, psycho-sociale et peut parfois impliquer le milieu scolaire. Enfin, et compte tenu du poids des troubles psychopathologiques chez les très jeunes suicidants, un suivi pédopsychiatrique sur une période minimale de un an est recommandé avec une psychothérapie et un accompagnement familial associé éventuellement au traitement pharmacologique de la pathologie psychiatrique [8]. L'alliance thérapeutique avec la famille est importante à instaurer afin d'améliorer la compliance au suivi. La comparaison des deux sous-groupes en fonction de l'âge n'a pas montré de différences significatives pour l'ensemble des caractéristiques recherchées en dehors du trouble de l'adaptation plus fréquent chez les moins de 13 ans, et la maltraitance plus fréquente chez les adolescents. Peyre et al ont comparé les primo-suicidants de moins de 13 ans versus les primo-suicidants âgés de plus que 13 ans, et ont retrouvé que les TS de l'enfant étaient associées à la maltraitance tandis que les TS de l'adolescent étaient associées à la présence d'un épisode dépressif majeur [6]. Cette différence peut être liée à la faible taille de notre échantillon, mais aussi à la difficulté de l'enfant de révéler la maltraitance au clinicien surtout quand celle-ci émane du milieu familial. L'utilisation de questionnaires ou d'échelles validés destinés aux enfants et aux adolescents permettra de mieux évaluer l'exposition à la maltraitance.

Limites: la principale limite de notre étude est liée à la taille réduite de notre échantillon, dans une perspective d'avenir, il serait important de faire des études transversales sur des échantillons plus larges. La deuxième limite concerne le biais de recrutement lié aux caractéristiques de la structure de soin où s'est déroulée l'étude, institution stigmatisé par la population, avec un nombre de cas de TS graves surreprésenté par rapport aux autres formes de TS. Enfin une autre limite découle des difficultés inhérentes aux entretiens réalisés dans un contexte d'urgence ne permettant pas de déterminer certains évènements de vie jugés comme tabous.

## Conclusion

Le passage à l'acte suicidaire est une forme fréquente de l'expression de la souffrance chez l'enfant et l'adolescent. En Tunisie, les études réalisées demeurent peu nombreuses. Les résultats de la présente étude ont apporté une meilleure connaissance du profil des enfants et adolescents suicidants. Ainsi, ces derniers ont confirmé la fréquence élevée de la dépression dans cette population, souvent réactionnelle au contexte environnemental ainsi que des antécédents de maltraitance retrouvés dans presque un cas sur deux, ce dernier est un facteur de risque reconnu par de nombreuses études. L'intentionnalité suicidaire est fréquemment élevée, et devrait être systématiquement évaluée par les soignants qu'ils soient urgentistes, pédiatres ou pédopsychiatres. La fréquence des signes de souffrance psychiques, des troubles du comportement et la survenue d'automutilations rapportés dans les semaines et les mois précédent le passage à l'acte suicidaires renforcent la nécessité de mettre en place des stratégies de prévention ciblées. Ces stratégies doivent permettre d'améliorer le dépistage des enfants qui présentent ces signaux d'alerte mais aussi des enfants à risque comme ceux exposés aux facteurs d'adversité psycho-sociaux et ceux qui présentent des troubles psychopathologiques. Dans le contexte tunisien, ce dépistage pourrait être envisagé le cadre de la médecine scolaire d'une part, et par les services sociaux et de protection de l'enfance -qui se sont beaucoup développés ces dernières années- d'autre part. De même qu'une sensibilisation des professionnels qui travaillent auprès des enfants et des adolescents devrait être développée afin de prévenir les comportements suicidaires et d'améliorer les facteurs de résiliences chez les sujets vulnérables.

### Etat des connaissances actuelle sur le sujet

- Les tentatives de suicide chez l'enfant et l'adolescent sont fréquentes et nécessitent une bonne évaluation des facteurs de risque;
- Plusieurs études ont confirmé le rôle des troubles mentaux en particulier les troubles dépressifs et des facteurs environnementaux psycho-sociaux dans l'émergence des comportements suicidaires à l'adolescence;
- La prise en charge psycho-sociale est souvent indiquée pour éviter la récidive.

### Contribution de notre étude à la connaissance

- La dépression est le trouble mental le fréquemment retrouvé chez l'enfant et l'adolescent suicidant;
- La maltraitance est un facteur de risque du suicide chez l'enfant et l'adolescent dans le contexte tunisien à l'instar de ce qui été retrouvé dans d'autres pays;
- L'intentionnalité suicidaire est fréquemment élevée et implique une évaluation du potentiel suicidaire chez l'enfant et l'adolescent après une tentative de suicide.

## Conflits d'intérêts

Les auteurs ne déclarent aucun conflit d'intérêts.
